# Influences of freezing–thawing actions on mechanical properties of soils and stress and deformation of soil slope in cold regions

**DOI:** 10.1038/s41598-022-09379-3

**Published:** 2022-03-30

**Authors:** Bo Xiang, Enlong Liu, Liuxin Yang

**Affiliations:** 1 Sichuan Provincial Transport Department Highway Planning, Survey, Design and Research Institute, Chengdu, 610041 Sichuan China; 2 State Key Laboratory of Hydraulics and Mountain River Engineering, College of Water Resources and Hydropower, Sichuan University, Chengdu, 610065 China; 3 State Key Laboratory of Frozen Soil Engineering, Northwest Institute of Eco-Environment and Resources, Chinese Academy of Sciences, Lanzhou, 730000 China

**Keywords:** Civil engineering, Natural hazards

## Abstract

Freezing–thawing actions can affect the mechanical features of soil greatly, which is vital for the stability of soil slope in cold regions. Firstly, triaxial compression tests on sand samples under undrained conditions were performed to investigate the influences of freezing–thawing cycles, which shows that the freezing–thawing actions can weaken their strength and stiffness, and with the increasing freezing–thawing cycles, both the deviatoric stress and pore water pressure decrease gradually. Then, the double hardening constitutive model was revised to model the influences of freezing–thawing cycles in consideration of the influences of freezing–thawing actions, and the model was also validated by the test results. Finally, the proposed constitutive model was incorporated into a finite element code to numerically simulate the distribution of displacement and pore water pressure of sand slope subjected to freezing–thawing cycles, which shows that the freezing–thawing actions accelerate the dissipation of the pore water pressure and enlarge the displacement of the slope. The study here can provide a help in designing and construction of civil engineering in cold regions.

## Introduction

In cold regions, soil mechanical properties will change greatly when subjected to the freezing–thawing (F–T) action, which can affect the design and construction of new projects and the stability and safety of the existing infrastructures^1^. Therefore, when designing projects to select soil parameters, and analyzing the stability and deformation, it is necessary to take into account the influences of F–T cycles on mechanical features of soils. With the rapid development of road and railway in Western China, some important projects have been constructed there, where soil ground is affected greatly by F–T actions, and thus study on the influences of F–T cycles on soils is very important and will be carried here.

Currently, many works have been done on the mechanical properties of soils experiencing F–T cycles, and it demonstrates that under the action of F–T, soil structure, density and void ratios will change, water contents will redistribute, and the limits of water contents are also affected; after thawing of frozen soils, their strength, pore water pressure and compressibility will change in some degree; and in the process of F–T actions, the variation of microstructures will change the permeability, which can vary a few orders of magnitude. Chamberlain and Gow in 1979^2^ performed laboratory tests to study the permeability and soil structure subjected to F–T cycles, and it found that under all the conditions, the F–T cycles can induce the decrease of porosity and the increase of permeability in the vertical direction. Parmdi et al. in 1996^3^ studied the influences of both F–T and drying-wetting cycles on soil structures and porosity through tests, and found that the F–T cycles can affect the porosity within surficial soil deposit and the entire soil structure greatly. Qi et al. in 2008^4^ investigated influences of F–T on physical and mechanical features of silty samples under different conditions, in which the variations of the dry weight, strength parameters, preconsolidated pressure, and modulus were analyzed, and found that there exists the critical dry weight for the same number of F–T cycles. Kamei et al. in 2012^5^ carried out tests on very soft clay soil stabilised with recycled bassanite underwent F–T cycles, and found that with the increasing F–T cycles, both the index of durability and strength of the samples. Aldaood et al. in 2013^6^ studied impact of freeze–thaw cycles on mechanical behaviour of lime stabilized gypseous soils, and found that the F–T cycles can reduce the unconfined strength. Shibi and Kamei in 2014^7^ performed tests on cement-stabilised soil containing recycled bassanite and coal ash, and studied the effect of freeze–thaw cycles on their strength and physical properties. Xie et al. in 2015^8^ carried out the freezing–thawing process of the soil samples extracted from the Qinghai-Tibet Plateau by laboratory experiments to determinate the volume variation of soil as well as physical and mechanical properties after soil experiences various freeze–thaw cycles, and the results demonstrated that cohesion and uniaxial compressive strength decreased as the volume and porosity of soil increased after experiencing various freeze–thaw cycles, especially in the first six freeze–thaw cycles. Qu et al. in 2019^9^ studied the effect of cyclic freeze–thaw on their uniaxial mechanical properties of cohesive coarse-grained soils extracted from a high-altitude slope in the Qinghai–Tibet Plateau, and the results showed that the stress–strain curves of the tested soils mainly behaved as strain-softening, and the uniaxial compressive strength, resilient modulus, residual strength and softening modulus decreased considerably with the increase of freeze–thaw cycles. Qi et al. in 2021^10^ carried out laboratory tests to quantitatively analyze the freeze–thaw effect on the soil engineering characteristics to reveal the facilitation on the bank slope instability, and the results show that the softening characteristics of the stress strain curves gradually weaken, the effective cohesions decline exponentially, the seepage coefficients enlarge, and the thermal conductivities decrease after 7 freeze–thaw cycles. Liu et al. in 2021^11^ performed a series of consolidated drained triaxial tests (CD) on the samples experienced various numbers of F–T cycles to study the evolution of mechanical and deformational behaviours of tailing soils experienced F–T cycles, and the experimental results indicated that the cementation failure and the expansion of the mean pore size inside tailings are the dominant factors those exert an adverse impact on the mechanical and deformation properties, based on which, a new constitutive model was also proposed.

Even though a lot of works have been done on influences of F–T on mechanical features of soil samples, there are still primary and more studies have to be carried out to understand the mechanical properties in the process of F–T cycles of soils. In order to study the influences of F–T cycles on mechanical properties of sand samples and the deformation and stress of slope, triaxial tests on sand samples are firstly conducted here, followed by revising the double hardening constitutive model to consider the influence of F–T actions, and finally, the constitutive model is numerically programmed via finite element method to simulate the stress and deformation of sand slope experiencing F–T cycles.

### Triaxial tests on soil samples experiencing freezing–thawing cycles

#### Test methods

**Figure 1:**
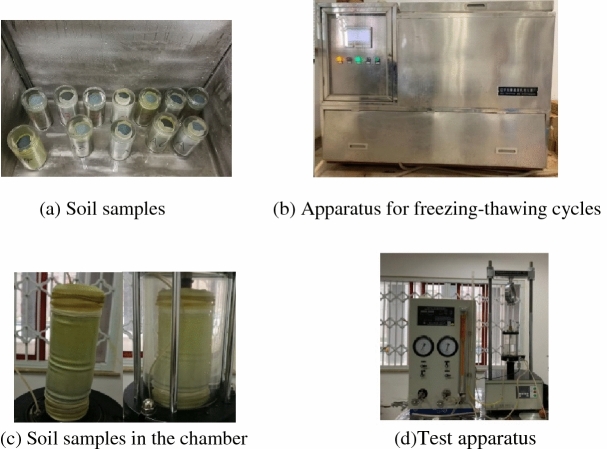
Freezing–thawing process of soil samples and the test apparatus.

The soil samples were extracted from a site of Gong-Ga Mountain in the southwest in China, with the altitude of roughly 2500 m, located at the superficial zone with a depth of 30–50 cm. The soil is classified as sand soil containing fine grains, with the natural water content 21.8%, and the particle sizes are 3.90% of 2.0–1.0 mm, 19.67% of 1.0–0.5, 32.20% of 0.5–0.25, 20.75% of 0.25–0.1, 10.05% of 0.1–0.075, and 13.43% smaller than 0.075. The tested soil samples were prepared with a layered compaction method according to the soil test methods, and the dry density is 1.28 g/cm^3^, 61.8 mm in diameter and 125 mm in height. Subsequently, the prepared samples were saturated by pumping air method, and for the samples without experiencing freezing–thawing cycles, they were directly put into the chamber of the triaxial apparatus for tests; but for those experiencing freezing–thawing actions, they were placed into the freeze-thawing apparatus, subjected to a freezing–thawing process, in which both the durations of freezing and thawing are 12 h, and the controlled cold temperature was −15 °C and the warm temperature 20 °C. For the sample experiencing freezing–thawing cycles, it was enclosed with a rubber membrane, then placed into a container and immersed in the water. One cycle of freezing–thawing is 24 h, and the samples underwent the freezing–thawing cycles 1, 5, and 10, respectively. Lastly, all the samples experiencing freezing–thawing cycles were also put into the chamber of the triaxial apparatus for tests. The tested samples were also saturated again by the back pressure in the triaxial chamber to assure the degree of saturation reaching 0.95. The samples were tested under consolidated undrained (CU) conditions at the confining pressure of 25, 100, 200, and 400 kPa respectively, and the loading rate controlled was 0.2 mm/min, with recording the axial displacement, deviatoric stress, and pore pressure in the entire process. Freezing–thawing process of soil samples and the test apparatus is shown in Fig. 1.

### Analysis on test results

**Figure 2:**
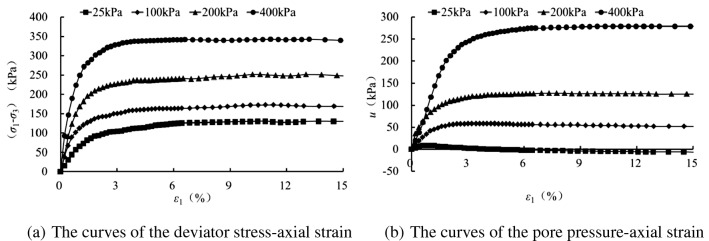
CU triaxial tested results of the samples of 0 freezing–thawing cycle.

**Figure 3:**
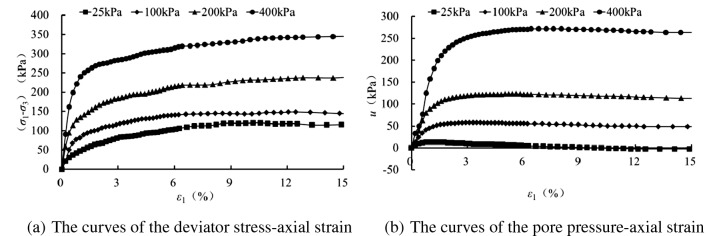
CU triaxial tested results of the samples of 1 freezing–thawing cycle.

**Figure 4:**
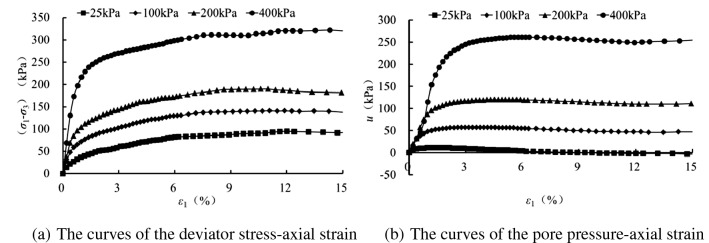
CU triaxial tested results of the samples of 5 freezing–thawing cycle.

**Figure 5:**
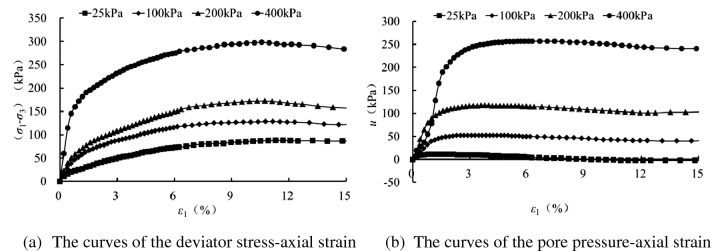
CU triaxial tested results of the samples of 10 freezing–thawing cycle.

Figures 2, 3, 4 and 5 present the deviatoric stress (${\sigma }_{1}-{\sigma }_{3}$) -axial strain and the pore pressure $u$—axial strain curves for the tested samples experiencing 0, 1 , 5 and 10 freezing–thawing cycles, which demonstrate that all the samples behave strain softening at different confining pressures and freeze-thawing cycles, and the pore pressure increases at the initial loading and then decreases obviously to a small value for the lower confining pressure, but it increases initially and tends to a stable value for the higher confining pressure. The higher the confining pressure, the higher the deviatoric stress and pore pressure, and this is due to that the soils tested are sandy soil having a relative larger grain, and the higher confining pressure can provide more resistance for the sample and also result in a higher water pore pressure under CU conditions.

**Figure 6:**
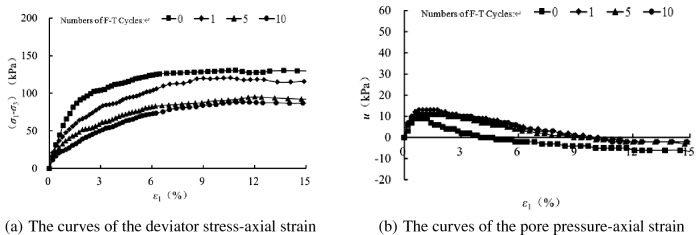
CU triaxial tested results of the samples at 25 kPa confining pressure.

**Figure 7:**
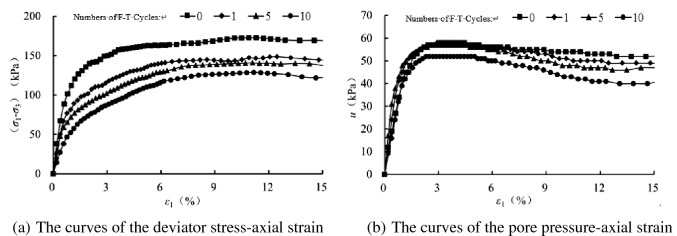
CU triaxial tested results of the samples at 100 kPa confining pressure.

**Figure 8:**
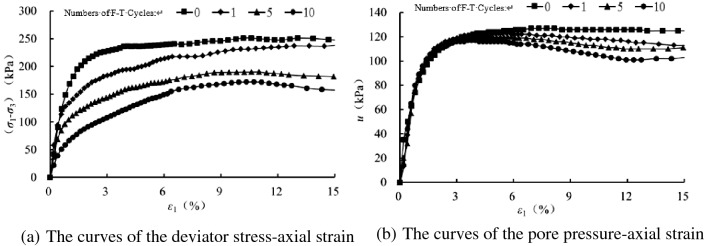
CU triaxial tested results of the samples at 200 kPa confining pressure.

**Figure 9:**
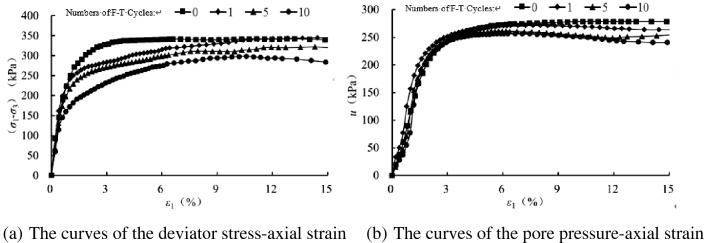
CU triaxial tested results of the samples at 400 kPa confining pressure.

In order to analyze the influence of freezing–thawing cycles on the mechanical properties of sand samples, we have compared the tested results with varying number so freezing–thawing cycles at the same confining pressure, as shown in Figs. 6, 7, 8 and 9. At the same confining pressure, with the increase of freezing–thawing cycles, both the deviatoric stress and the maximum positive value of the pore pressure becomes smaller, but at 25 kPa confining pressure the maximum positive value of the pore pressure becomes bigger with increasing freezing–thawing cycles. The reason for this is that: the soil is sand soil, and it behaves contracts firstly, followed by dilatancy under drained conditions, so under undrained conditions, the positive pore water pressure increases firstly and then becomes negative due to the constant volumetric strain. With the increasing number of freezing–thawing cycles, the structure formed by the freezing–thawing process affects the development of the pore pressure more greatly. At 25 kPa confining pressure, when the consolidation process finishes, soil structure is almost not damaged, so the positive pore pressure increases with the increasing number of freezing–thawing cycles. But at 100 kPa or higher confining pressure, soil structure can be almost damaged at the end of consolidation process, so the pore water pressure has a different changing rule.

During the freezing–thawing process, the formation of ice crystals in the pores will enlarge the samples and thus it cannot be recovered totally when the ice crystals melt, which thus results the decrease of the soil strength. When the confining pressure is small, the sand sample has the tendency of dilatancy, which is restrained by the generation of the negative pore pressure, as shown in Fig. 6 of 25 kPa confining pressure. But, with the increasing confining pressure, the sand sample tends to contract, and thus the positive pore pressure is produced.

### Constitutive model for soil considering freezing–thawing cycles

#### Constitutive model

In order to simulate the stress–strain relationship of the sand samples experiencing freezing–thawing process, an elasto-plastic constitutive model is proposed here, in which two hardening parameters are employed. The yielding function has the following expression ^12^:

1 $$F=\frac{{\sigma }_{m}^{\mathrm{^{\prime}}}}{1-{\left(\frac{\eta }{\alpha }\right)}^{n}}-p=0$$

in which ${\sigma }_{m}^{\mathrm{^{\prime}}}=\frac{1}{3}\left({\sigma }_{1}^{\mathrm{^{\prime}}}+{\sigma }_{2}^{\mathrm{^{\prime}}}+{\sigma }_{3}^{\mathrm{^{\prime}}}\right)$,$\eta =\frac{{\sigma }_{s}}{{\sigma }_{m}^{\mathrm{^{\prime}}}}$,${\sigma }_{s}=\frac{1}{\sqrt{2}}{\left[{\left({\sigma }_{1}^{\mathrm{^{\prime}}}-{\sigma }_{2}^{\mathrm{^{\prime}}}\right)}^{2}{+\left({\sigma }_{2}^{\mathrm{^{\prime}}}-{\sigma }_{3}^{\mathrm{^{\prime}}}\right)}^{2}{+\left({\sigma }_{3}^{\mathrm{^{\prime}}}-{\sigma }_{1}^{\mathrm{^{\prime}}}\right)}^{2}\right]}^{1/2}$,$n$ is constant, $p$ and $\alpha$ are two hardening parameters. For the parameter $p$, which reflects the influence of plastic volumetric strain, can be expressed as

2 $$p={p}_{0}\mathrm{exp}\left(\frac{{\varepsilon }_{v}^{p}}{{c}_{c}-{c}_{s}}\right)$$

in which ${\varepsilon }_{v}^{p}={\varepsilon }_{1}^{p}{+\varepsilon }_{2}^{p}{+\varepsilon }_{3}^{p}$, ${p}_{0}$ is the reference pressure when ${\varepsilon }_{v}^{p}=0$, ${c}_{c}=\frac{\lambda }{1+{e}_{0}}$, ${c}_{s}=\frac{\kappa }{1+{e}_{0}}$, and $\lambda$ and $\kappa$ are respectively the slopes of compression and rebound curves in $e\sim \mathrm{ln}p$ plane, ${e}_{0}$ is the initial void ratio.

3 $$\alpha ={\alpha }_{m}-{\alpha }_{0}\mathrm{exp}\left(\frac{{\varepsilon }_{s}^{p}}{{c}_{a}}\right)$$

The parameter $\alpha$ is the function of the generalized shear strain, ${\varepsilon }_{s}^{p}=\frac{1}{\sqrt{2}}{\left[{\left({\varepsilon }_{1}^{p}-{\varepsilon }_{2}^{p}\right)}^{2}{+\left({\varepsilon }_{2}^{p}-{\varepsilon }_{3}^{p}\right)}^{2}{+\left({\varepsilon }_{3}^{p}-{\varepsilon }_{1}^{p}\right)}^{2}\right]}^{1/2}$, ${\alpha }_{m}$, ${\alpha }_{0}$ and ${c}_{a}$ are the model parameters, which are affected by the freezing–thawing cycles. According to the associated flow rule, namely $f=g$, the increments of ${\varepsilon }_{v}^{p}$ and ${\varepsilon }_{s}^{p}$ can be obtained as follows,

4 $${d\varepsilon }_{v}^{p}=\frac{1}{H}\left(\frac{\partial F}{\partial {\sigma }_{m}^{\mathrm{^{\prime}}}}\frac{\partial F}{\partial {\sigma }_{m}^{\mathrm{^{\prime}}}}d{\sigma }_{m}^{\mathrm{^{\prime}}}+\frac{\partial F}{\partial {\sigma }_{s}}\frac{\partial F}{\partial {\sigma }_{m}^{\mathrm{^{\prime}}}}d{\sigma }_{s}\right)$$

5 $${d\varepsilon }_{s}^{p}=\frac{1}{H}\left(\frac{\partial F}{\partial {\sigma }_{m}^{\mathrm{^{\prime}}}}\frac{\partial F}{\partial {\sigma }_{s}}d{\sigma }_{m}^{\mathrm{^{\prime}}}+\frac{\partial F}{\partial {\sigma }_{s}}\frac{\partial F}{\partial {\sigma }_{s}}d{\sigma }_{s}\right)$$

in which $H$ is the hardening modulus, and expressed as

6 $$H=-\frac{\partial F}{\partial p}\frac{\partial p}{\partial {\varepsilon }_{v}^{p}}\frac{\partial F}{\partial {\sigma }_{m}^{\mathrm{^{\prime}}}}-\frac{\partial F}{\partial \alpha }\frac{\partial \alpha }{\partial {\varepsilon }_{s}^{p}}\frac{\partial F}{\partial {\sigma }_{s}}$$

We also have $\frac{\partial F}{\partial {\sigma }_{m}^{\mathrm{^{\prime}}}}=\frac{1-\left(1+n\right){\left(\eta /\alpha \right)}^{n}}{{\left[1-{\left(\eta /\alpha \right)}^{n}\right]}^{2}}$, $\frac{\partial F}{\partial {\sigma }_{s}}=\frac{n{\left(\eta /\alpha \right)}^{n-1}}{{\left[1-{\left(\eta /\alpha \right)}^{n}\right]}^{2}}$, $\frac{\partial F}{\partial p}=-1$, $\frac{\partial F}{\partial \alpha }=-\frac{n{\sigma }_{m}^{\mathrm{^{\prime}}}{\left(\eta /\alpha \right)}^{n}}{\alpha {\left[1-{\left(\eta /\alpha \right)}^{n}\right]}^{2}}$, $\frac{\partial p}{\partial {\varepsilon }_{v}^{p}}=\frac{p}{{c}_{c}-{c}_{s}}$, $\frac{\partial \alpha }{\partial {\varepsilon }_{s}^{p}}=\frac{\alpha -{\alpha }_{m}}{{c}_{a}}$.

The elastic strain can be computed as follows,

7 $${d\varepsilon }_{v}^{e}=\frac{d{\sigma }_{m}^{\mathrm{^{\prime}}}}{K}$$

8 $${d\varepsilon }_{s}^{e}=\frac{d{\sigma }_{s}}{3G}$$

in which $K=\frac{E}{3\left(1-2\nu \right)}$, $G=\frac{E}{2\left(1+\nu \right)}$, $E$ is the elastic modulus, and $\upsilon$ is the Possion ratio.

Summing Eqs. (4) and (7), and Eqs. (5) and (8) respectively, the total increments of volumetric and shear strain are obtained as follows,

9 $$d{\varepsilon }_{v}={d\varepsilon }_{v}^{e}+{d\varepsilon }_{v}^{p}=\frac{d{\sigma }_{m}^{\mathrm{^{\prime}}}}{K}+\frac{1}{H}\left(\frac{\partial F}{\partial {\sigma }_{m}^{\mathrm{^{\prime}}}}\frac{\partial F}{\partial {\sigma }_{m}^{\mathrm{^{\prime}}}}d{\sigma }_{m}^{\mathrm{^{\prime}}}+\frac{\partial F}{\partial {\sigma }_{s}}\frac{\partial F}{\partial {\sigma }_{m}^{\mathrm{^{\prime}}}}\mathrm{d}{\sigma }_{s}\right)$$

10 $$d{\varepsilon }_{s}={d\varepsilon }_{s}^{e}+{d\varepsilon }_{s}^{p}=\frac{d{\sigma }_{s}}{3G}+\frac{1}{H}\left(\frac{\partial F}{\partial {\sigma }_{m}^{\mathrm{^{\prime}}}}\frac{\partial F}{\partial {\sigma }_{s}}d{\sigma }_{m}^{\mathrm{^{\prime}}}+\frac{\partial F}{\partial {\sigma }_{s}}\frac{\partial F}{\partial {\sigma }_{s}}d{\sigma }_{s}\right)$$

### Determination of model parameters and validation

The parameters in the model include elastic and plastic parts, and they are $K$,$G$, $n$, ${c}_{c}$, ${c}_{s}$, ${\alpha }_{m}$, ${\alpha }_{0}$, ${c}_{a}$. For the elastic modulus *E*, it can be determined approximately within 0.2% of the axial strain, and for the different F-T cycles it can be determined by ${\sigma }_{3}$, as shown in Table 1. After assuming $\nu =0.2$, $K=\frac{E}{3\left(1-2\upsilon \right)}$ and $G=\frac{E}{2\left(1+\upsilon \right)}$ can be obtained. The shape of the yielding surface can be reflected by *n*, which is assumed to be $1.3$ here. According to ${c}_{c}=\frac{\lambda }{1+{e}_{0}}$, ${c}_{s}=\frac{\kappa }{1+{e}_{0}}$, we know that once the initial void ratio ${e}_{0}$, the slope $\lambda$ of $e\sim \mathrm{ln}p$ compression curve, and $\kappa$ of $e\sim \mathrm{ln}p$ rebound curve, ${c}_{c}$ and ${c}_{s}$ can be determined. Through the isotropic compression curves of the sand samples subjected to freezing–thawing cycles, we can obtain the values of ${e}_{0}$, ${c}_{c}$, ${c}_{s}$, and ${e}_{0}=0.04\mathrm{ln}\left(N+1\right)+1.1$, $\lambda =0.001\left(N+1\right)+0.06$, N is the number of F-T cycles and $\kappa =0.009$. ${\alpha }_{m}$, ${\alpha }_{0}$, ${c}_{a}$ are affected by the freezing–thawing process stress level, which can be determined according to the following expressions: ${\alpha }_{m}=\left(0.0257\frac{{\sigma }_{3}}{{p}_{a}}-0.3377\right)\mathrm{ln}\left(N+0.1\right)-0.3032\frac{{\sigma }_{3}}{{p}_{a}}+4.7915$, ${\alpha }_{0}=\left(0.614\frac{{\sigma }_{3}}{{p}_{a}}-0.3515\right)\mathrm{ln}\left(N+0.1\right)-0.5122\frac{{\sigma }_{3}}{{p}_{a}}+4.2892$, and ${c}_{a}=\left(-0.0008\frac{{\sigma }_{3}}{{p}_{a}}+0.00004\right)\mathrm{ln}\left(N+0.1\right)-0.002\frac{{\sigma }_{3}}{{p}_{a}}-0.0048$, in which ${p}_{a}$ is the standard air pressure, and N is the number of F–T cycles.

**Table 1:** Elastic modulus.

F-T cycles	Confining pressure (kPa)	Values of E (kPa)	E (kPa)
0	25	7529.56	$E=98{\sigma }_{3}+4069$
	100	14,673.08	
	200	20,685.67	
	400	44,653.46	
1	25	10,523.22	$E=94{\sigma }_{3}+8537$
	100	17,172.66	
	200	29,022.85	
	400	45,318.12	
5	25	7305.90	$E=71{\sigma }_{3}+5774$
	100	14,072.25	
	200	18,739.57	
	400	34,650.70	
10	25	5675.64	$E=68{\sigma }_{3}+1326$
	100	7414.42	
	200	11,021.77	
	400	30,485.60	

**Figure 10:**
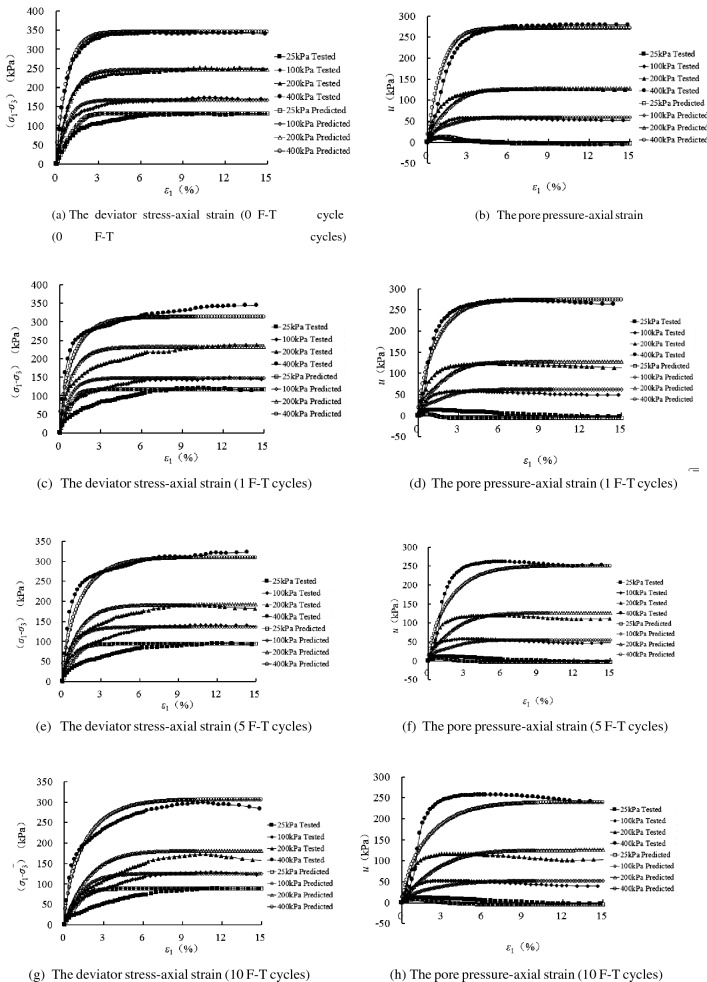
Comparisons of tested and predicted results of 10 freezing–thawing cycles.

According to the model parameters determined above, the comparisons of tested and predicted results for the different freezing–thawing cycles are shown in Fig. 10. From these figures, we know that the proposed model can grasp the main mechanical properties of the sand samples experiencing freezing–thawing cycles, and with the increasing confining pressure the deviatoric stress increases and the pore pressure increases initially, followed by decreases at the lower confining pressure, and increases all the time at the higher confining pressure. The influence of freezing–thawing process on the mechanical features of sand samples can also be duplicated.

### Numerical analysis of stress and deformation on soil slope subjected to thawing–thawing actions

#### Computation method and slop model experiencing F–T actions

The computed method is the finite element method, in which Biot equations for consolidation are solved numerically. The macroscopic hydromechanical behavior of saturated sand slope can be described as governed by a static force equilibrium:

11 $$\nabla \cdot {\varvec{\sigma}}+\rho \mathbf{g}=0$$

where ${\varvec{\sigma}}={\varvec{\sigma}}\mathrm{^{\prime}}-\mathrm{Ip}$, ${\varvec{\sigma}}\mathrm{^{\prime}}$ is the effective stress tensor, and the constitutive equation written in the following incremental form as:

12 $$d{\varvec{\sigma}}={\varvec{D}}:d{\varvec{\varepsilon}}-\mathrm{Ip}$$

where $\mathrm{I}$ is the identity tensor, ${\varvec{D}}$ is the stiffness tensor of incremental elasto-plastic constants. For the constitutive equations of Eqs. (9) and (10), the components of ***D*** can be expanded in Cartesian coordinates as:

13 $$\left[{\varvec{D}}\right]=\left[\begin{array}{cc}{D}_{11}& {D}_{12}\\ {D}_{21}& {D}_{22}\end{array}\right]$$

in which $${D}_{11}=\frac{1}{K}+\frac{1}{H}\frac{\partial F}{\partial {\sigma }_{m}^{\mathrm{^{\prime}}}}\frac{\partial F}{\partial {\sigma }_{m}^{\mathrm{^{\prime}}}}$$, $${D}_{12}=\frac{1}{H}\frac{\partial F}{\partial {\sigma }_{s}}\frac{\partial F}{\partial {\sigma }_{m}^{\mathrm{^{\prime}}}}$$, $${D}_{21}=\frac{1}{H}\frac{\partial F}{\partial {\sigma }_{m}^{\mathrm{^{\prime}}}}\frac{\partial F}{\partial {\sigma }_{s}}$$, $${D}_{22}=\frac{1}{3G}+\frac{1}{H}\frac{\partial F}{\partial {\sigma }_{s}}\frac{\partial F}{\partial {\sigma }_{s}}$$.

Application of the standard finite element discretization to the governing equations and boundary condition equations and approximating the variation of displacement **u** and pore pressure *p* by the nodal values and shape functions as

14 $$\mathbf{u}=\mathrm{N}\overline{\mathbf{u} }, p=\overline{\mathrm{N} }\overline{\mathrm{p} }$$

where $\mathrm{N}$ and $\overline{\mathrm{N} }$ are the shape functions for the displacement and the pore pressure respectively. According to reference^13^, the finite element equations can be solved numerically by discretizing in both space and time domains.

**Figure 11:**
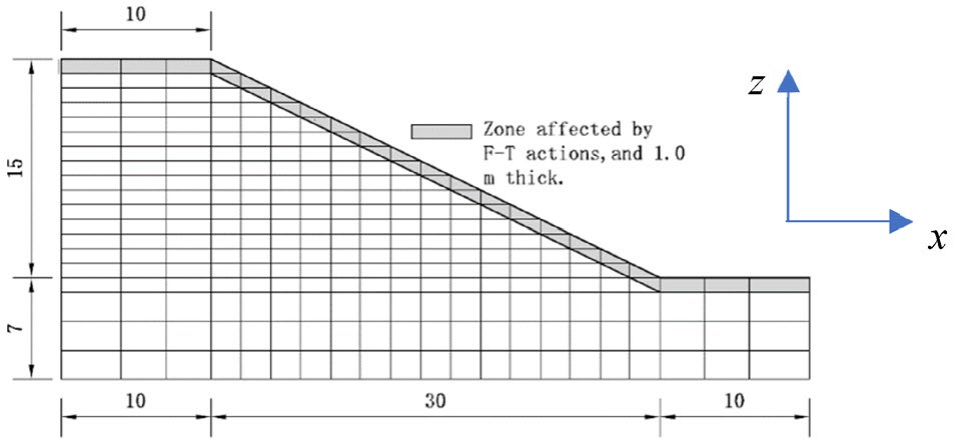
Computed mesh of saturated sand slope with 1 m thick soil layer subjected to F–T actions (unit of length: m).

The computed mesh for saturated sand slope subjected to F-T actions is shown in Fig. 11, in which 1.0 m thick surface soil layer is considered to undergo freezing–thawing actions, and the soil slope is 15 m in height and 30 m in width. The boundary conditions are that only the upper surface drains and the other boundaries are undrained, and the displacement is fixed at the bottom, at both right and left sides the horizontal displacement is fixed, and the displacement at other surfaces are free. The top surface is loaded 200 kPa within a short duration, with time increases to 20 years, the pore water pressure will dissipate gradually and displacement also changes, which can be simulated by this method, and the influences of freezing–thawing can also be obtained. The computed parameters of the constitutive model for the sand soil slope can be found in the last section and the permeabilities are 10^–6^ cm/s for soil without freeze-thawing process and 10^–3^ cm/s for 5 F–T, which is due to that the F–T process usually enlarges the soil permeability.

**Figure 12:**
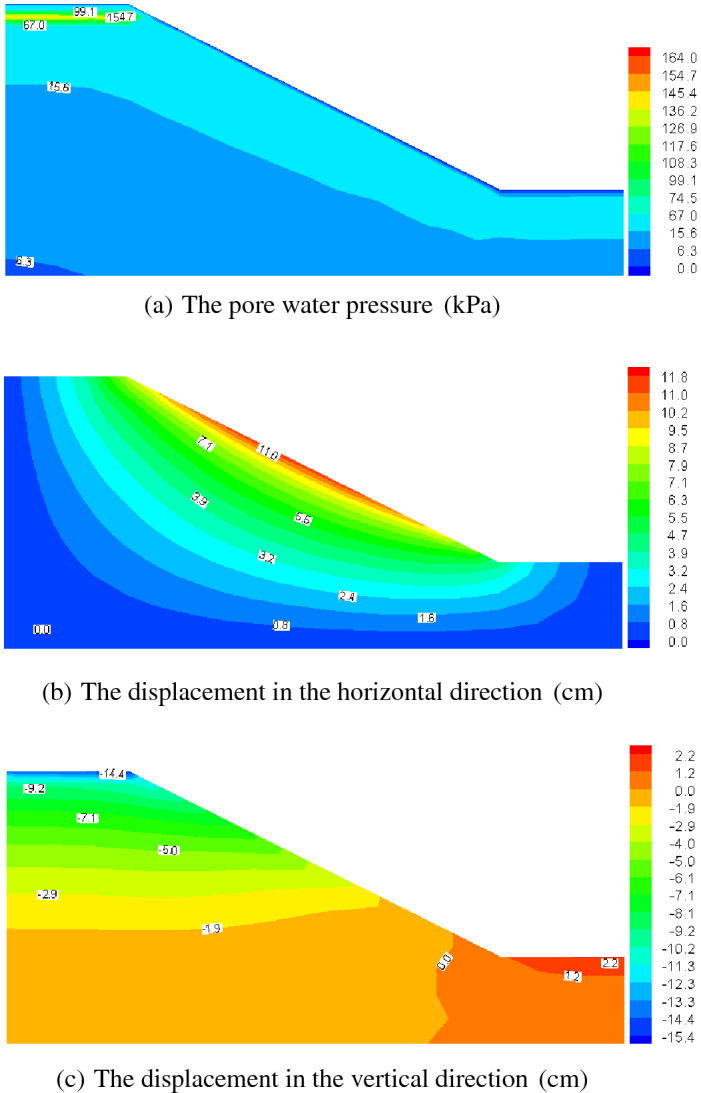
Computed results of 0 F–T cycles (initial loading).

**Figure 13:**
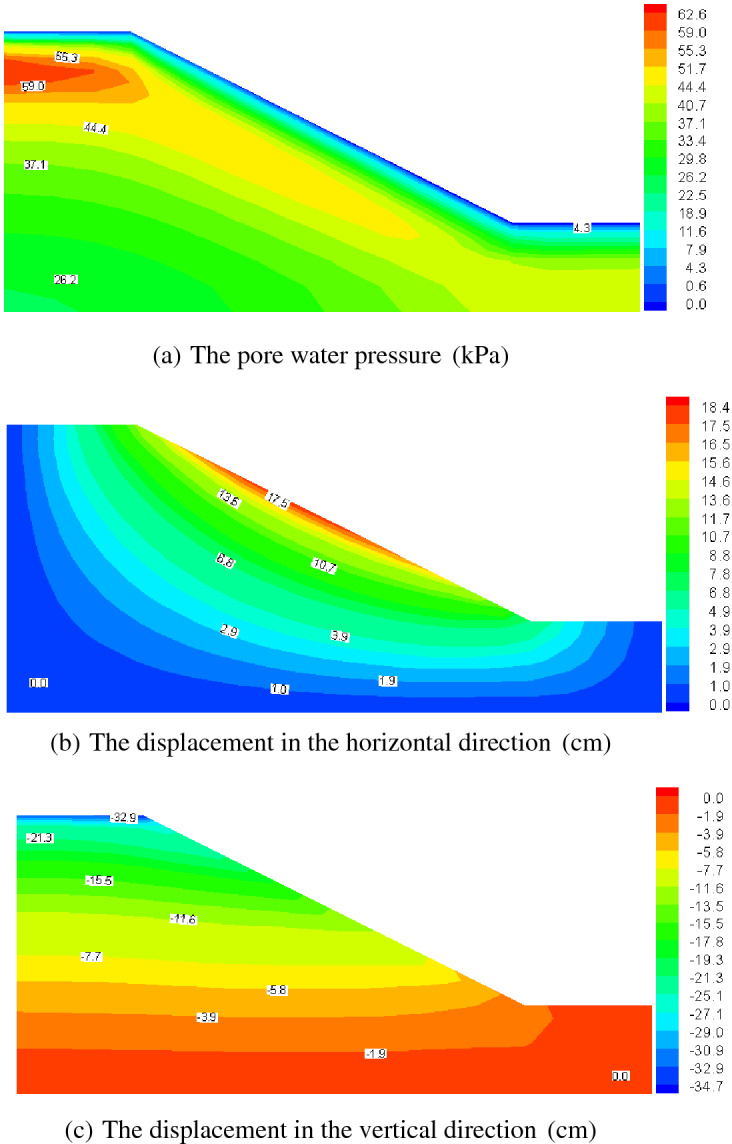
Computed results of 0 F–T cycles (20 years).

**Figure 14:**
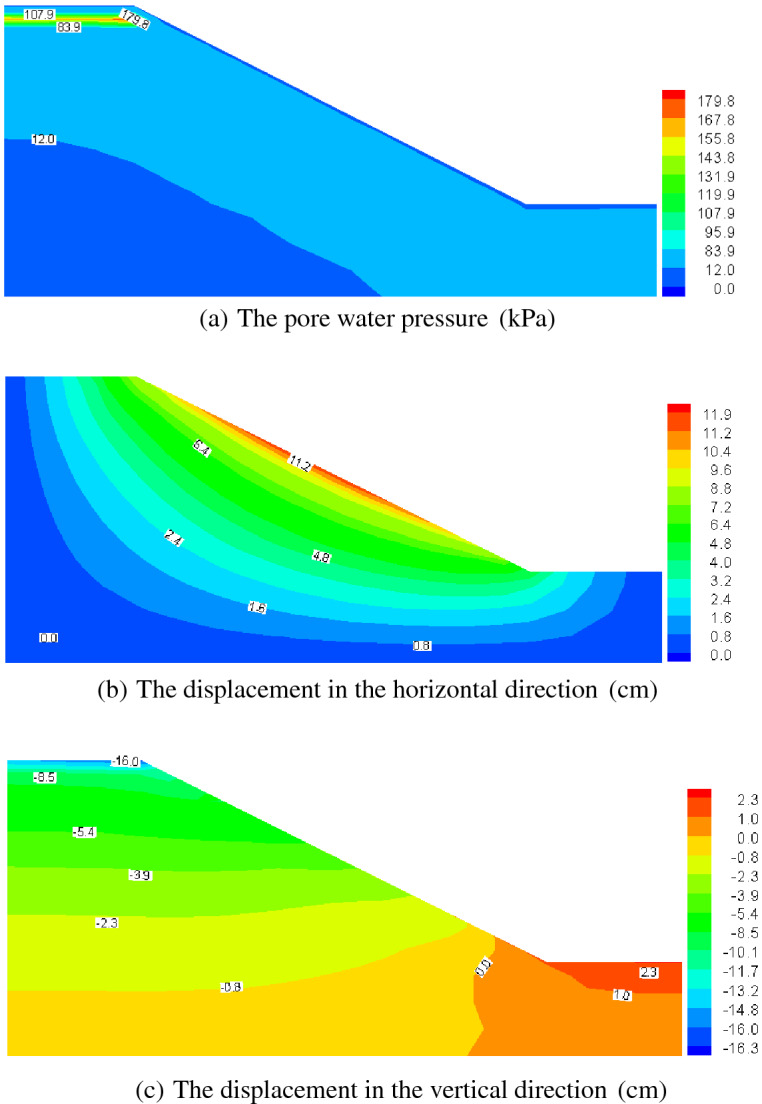
Computed results of 5 F–T cycles (initial loading).

**Figure 15:**
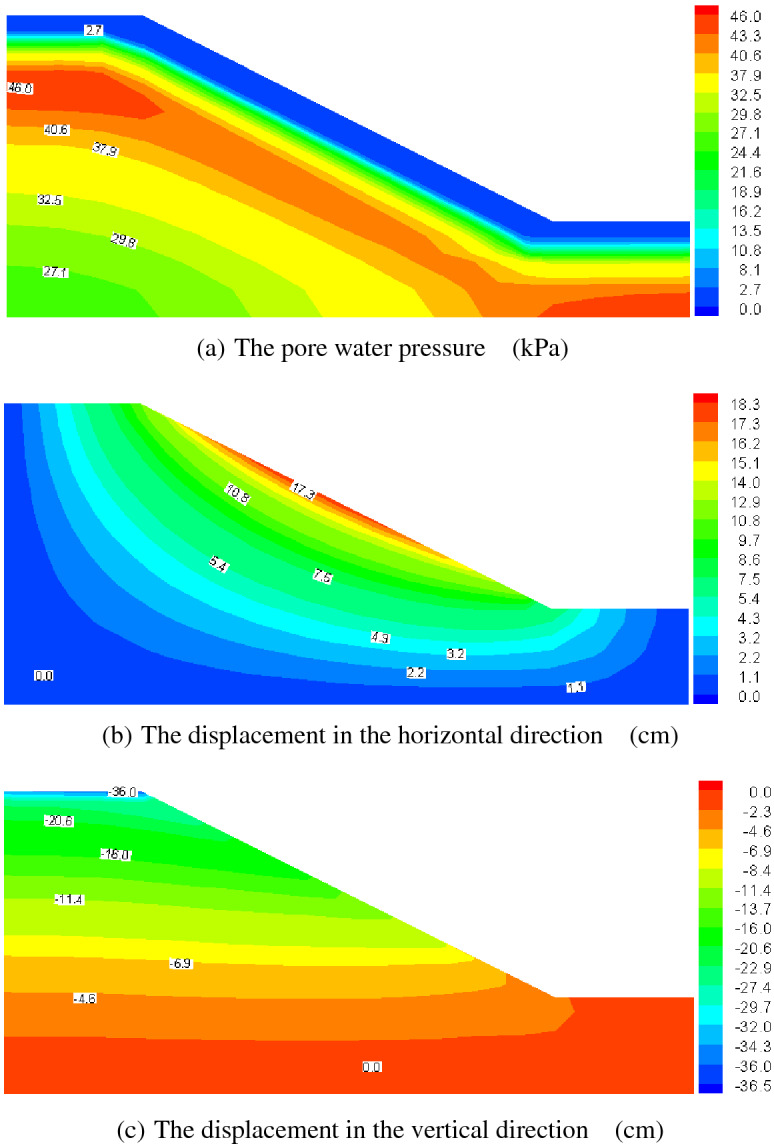
Computed results of 5 F–T cycles (20 years).

Figures 12, 13, 14 and 15 present the computed results for the sand slope subjected to 0 and 5 F–T cycles at the initial loading and 20 years. Initially, the pore water is bigger in the upper zone of the slope, and with the load of 200 kPa remaining constant, the pore water pressure dissipates and decreases to a lower value of 62.6 kPa in Fig. 13a lasting 20 years for the soil without F–T action, and the vertical displacement also increases due to the effective stress becoming larger. The freezing–thawing actions affect the pore water pressure and displacement greatly, which can be understood by comparing Figs. 12 and 14, and Figs. 13 and 15. Due to the strength weakening and the permeability enlarging with the increasing cycles of F–T, the values of displacement increase and the pore water pressure dissipates quickly. At 20 years of loading, the maximum value of the pore water pressure is 46.0 kPa for 5 cycles of F–T, which is smaller than that value of 62.6 kPa for soil without F–T action.

## Discussions

In this section, some discussions will be given on freezing–thawing pattern on the variation of soil sample volume, the potential influences of confining pressures to freezing–thawing actions on soil samples, and the influences of freezing–thawing cycles on slope stability.

For the testing procedure, the soil samples were subjected to freezing–thawing cycles in the apparatus first, then put in the chamber of triaxial apparatus, as shown in Fig. 1. The soil samples dilated slightly after the freezing–thawing cycles, for example, for 1 freezing–thawing cycle, the volume of soil sample increased by 2.9%, for 5 freezing–thawing cycle, the volume of soil sample increased by 5.6%. The reason for this is that: when soil samples are frozen, ice crystals form and more water is migrated into the sample, so the sample becomes larger in volume, and when temperature increases, ice crystals melt but the pore structure can be kept, so the samples experiencing freezing–thawing cycles can become larger slightly in volume.

For the sample experiencing freezing–thawing cycles, it was enclosed with a rubber membrane, then placed into a container and immersed in the water. So during the process of freezing–thawing, the rubber membrane has lateral confined pressure in some degree on the sample. Occurring to this effect, the area of the sample experiencing freezing–thawing cycles hardly changed, for 1, 5 and 10 freezing–thawing cycles, the area reduces by 0.7%, 0.8%, and 2.2% respectively. If the lateral displacement was kept constant, the height of the soil sample experiencing freezing–thawing cycles may become larger, and soil samples have large volumetric expansion. In the future study, we will investigate this.

Table 2 presents the maximum value of displacement and pore pressure, from which we can draw the conclusions that the slope stability is reduced. This reason for this is that: at initial loading, the pore water pressure is larger for soil slope underwent 5 F–T cycles, which will lead to a smaller effective stress and decline the stability; and with the development of consolidation process, the displacement becomes larger for soil slope underwent 5 F–T cycles, which is also an indicator of stability and thus the soil slope experiencing freezing–thawing actions has a less stability. In view of analysis mentioned above, when designing soil slope in cold regions, we should take some engineering measures to avoid the superficial soil mass underwent freezing–thawing actions.

**Table 2:** The maximum value of displacement and pore pressure.

F–T cycles	Time	Pore pressure(kPa)	Horizontal displacement (cm)	Vertical displacement (cm)
0	Initial loading	164.0	11.816	15.430
	20 years	62.6	18.492	34.787
5	Initial loading	179.8	11.964	16.326
	20 years	46.0	18.332	36.581

## Conclusions

The influences of freezing–thawing actions on mechanical features of sand samples under undrained conditions and the distributions of displacement and pore water pressure of sand slope are investigated in this study through triaxial tests, analysis in theory and numerical simulation. The conclusions can be summarized as follows.

- (i) The freezing–thawing actions affect the mechanical features of sand samples under undrained conditions greatly, which weakens their stiffness and strength, and leads the sand slope to have a bigger displacement and quicker dissipation of the pore water pressure.
- (ii) For the sand samples experiencing the F–T actions, with the increase of freezing–thawing cycles, the deviatoric stress decreases gradually and the pore water pressure becomes smaller at the same confining pressure. This is due to that freezing–thawing actions enlarge the pores within the samples resulted by formation and melting of ice crystals.
- (iii) Introducing the influences of freezing–thawing actions into the model parameters, the double hardening constitutive model can be employed to describe the stress–strain relations of sand samples. Compared with test results, the proposed model can duplicate the salient features of sand samples experiencing freezing–thawing actions.
- (iv) The constitute model proposed in this study is incorporated into the finite element procedure to perform the coupling analysis of deformation and seepage of the sand slope subjected to freezing–thawing, and the simulated results demonstrate that the freezing–thawing actions speed the dissipation of the pore water pressure and enlarge the displacement of the slope.
